# PReS-FINAL-2071: Anti-type II collagen antibodies, anti-CCP, IgA-RF and IgM-RF are associated with joint damage eight years after onset of juvenile idiopathic arthritis (JIA)

**DOI:** 10.1186/1546-0096-11-S2-P83

**Published:** 2013-12-05

**Authors:** L Berntson, K Aalto, A Fasth, T Herlin, S Nielsen, E Nordal, M Rygg, J Rönnelid

**Affiliations:** 1 Dept of Paediatrics, Uppsala University Hospital, Uppsala, Sweden; 2 Dept of Paediatrics, Helsinki University Hospital, Helsinki, Finland; 3 Dept of Paediatrics, University of Gothenburg, Gothenburg, Sweden; 4 Dept of Paediatrics, Århus University Hospital, Århus, Denmark; 5 Pediatric Rheumatology Department, Pediatric Clinic II, Copenhagen University Hospital, Copenhagen, Denmark; 6 Dept of Paediatrics, University Hospital of North Norway, Tromsø, Tromsø, Norway; 7 Department of Laboratory Medicine, Children's and Women's Health, and Department of Pediatrics, Norwegian University of Science and Technology and St.Olav's Hospital, Trondheim, Norway; 8 Department of Immunology, Genetics and Pathology, Uppsala University, Uppsala, Sweden

## Introduction

Antibodies specific for native human type II collagen (anti-CII) characterize an early inflammatory/destructive phenotype in adults with RA. There are no data presented so far on this antibody and its possible influence on disease course/outcome in children with JIA.

## Objectives

We wanted to relate occurrence of anti-CII, IgM-RF, IgA-RF and anti-CCP to assessment of joint damage, outcome and ILAR categories after eight years of disease in children with JIA.

## Methods

From the Nordic JIA database 192 patients with available sera were included. Serum samples were collected at a median time of 4 months (IQR 2-7) after disease onset. Patients were followed prospectively for a median of 97 months (IQR 95-105). At the 8-year follow-up visit, data on remission according to the preliminary criteria of C. Wallace et al as well as joint damage according to JADI were collected. Frozen and stored sera were analyzed with enzyme immunoassays for anti-CII, IgM-RF, IgA-RF and anti-CCP. Reference values for adults were used.

## Results

Anti-type II collagen antibodies occurred in 3.1% of patients sera, IgM-RF in 3.6%, IgA-RF in 3.1%, anti-CCP in 2.6% of the patients. Occurrence of RF and anti-CCP did to some extent overlap, but rarely with anti-CII. The polyarticular and oligoarticular extended categories were overrepresented in patients with any of the four autoantibodies. All four autoantibodies were significantly associated with joint damage after eight years, but we found no association with ongoing disease activity after eight years.

## Conclusion

- Occurrence of anti-CII did rarely overlap with anti-CCP, IgA-RF and IgM-RF, but all four were associated with joint damage after 8 years of disease in patients with JIA.

- Further studies on anti-CII in patients with JIA are needed.

## Disclosure of interest

None declared.

